# Supplementary material for: Analysis of the Human Protein Atlas Weakly Supervised Single-Cell Classification competition
Source: Nat Methods. 2022 Sep 29;19(10):1221–9. doi: 10.1038/s41592-022-01606-z (PMC9550622; doi:10.1038/s41592-022-01606-z)
Supplement: Supplementary file 1 — Supplementary Figs. 1–10, Supplementary Notes 1–5 [file 41592_2022_1606_MOESM1_ESM.pdf]

nature portfolio

https://doi.org/10.1038/s41592-022-01606-z

Supplementary information

Analysis of the Human Protein Atlas Weakly Supervised Single-Cell Classification competition

In the format provided by the authors and unedited

Supplementary Information for Analysis of the Human Protein Atlas Weakly-Supervised Single Cell Classification Competition

Supplementary Figure 1. Association between cell count/segmentation score and average precision score

Supplementary Figure 2. Score progression

Supplementary Figure 3. Violin plot of the score distribution per label for the top 50 teams

Supplementary Figure 4. Team 1 (bestfitting) - Solution summary and model architecture

Supplementary Figure 5. Team 1 (bestfitting) - Comparison between CNN, Puzzle-CAM and FCAN

Supplementary Figure 6. Team 2 ([red.ai]) - Solution summary and model architecture

Supplementary Figure 7. Team 3 (MPWARE & ZFTurbo & Dieter) - Solution summary and model architecture

Supplementary Figure 8. Team 4 - Solution summary and model architecture

Supplementary Figure 9. Class attention and green channel: intersection over union

Supplementary Figure 10. Subtle single cell heterogeneity

Supplementary Notes 1. Team 1 - bestfitting

1.1 Model description

1.2 Ablation study

1.3 Conclusion

Supplementary Notes 2. Team 2 - [red.ai]

2.1 Model description

2.2 Ablation study

2.3 Conclusion

Supplementary Notes 3. Team 3 - MPWARE & ZFTurbo & Dieter

3.1 Model description

3.2 Ablation study

3.3 Conclusion

Supplementary Notes 4. Team 4 - MILIMED

4.1 Model description

4.2 Ablation study

4.3 Conclusion

Supplementary Notes 5. Method summary

References

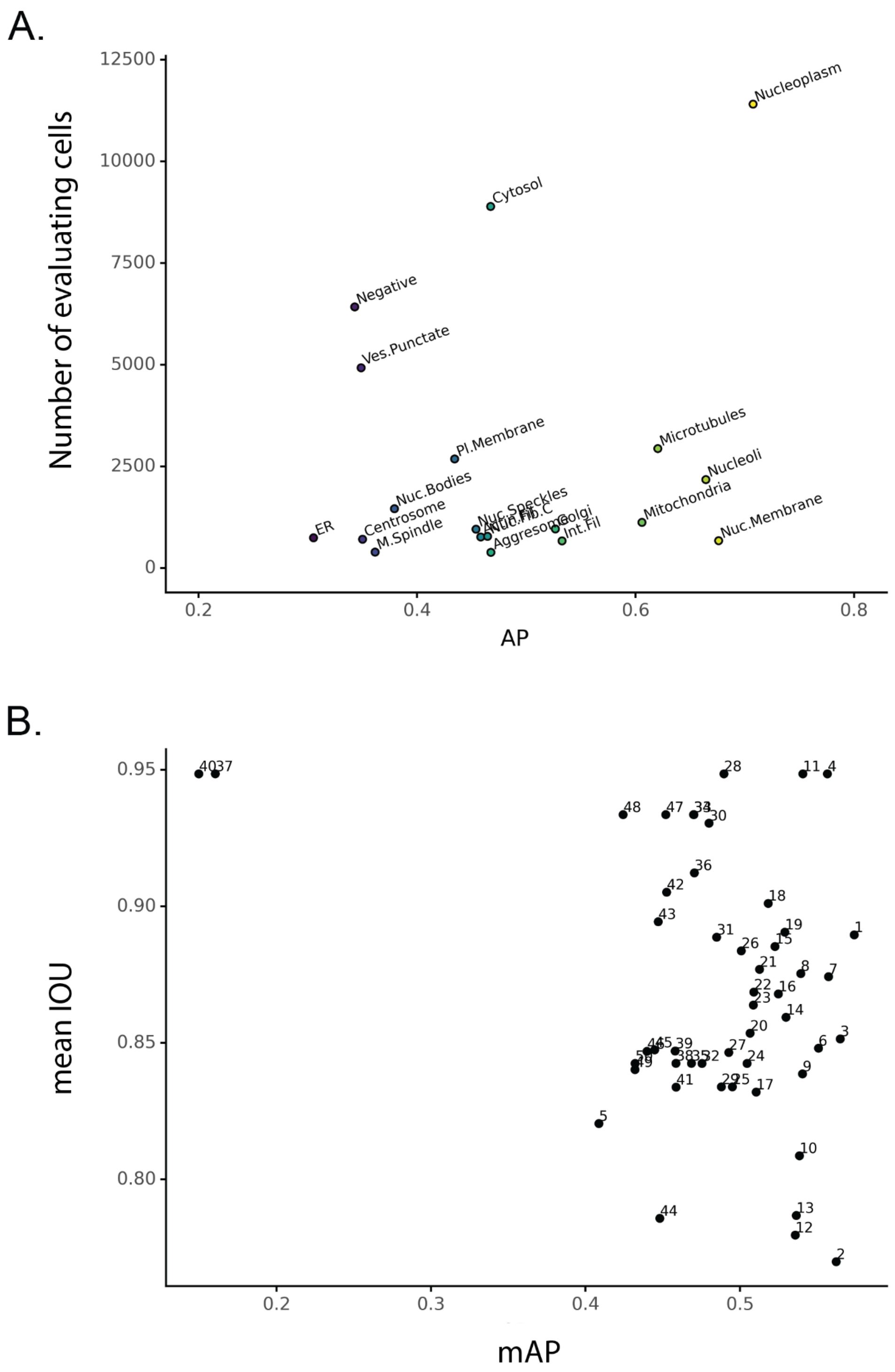

Supplementary Figure 1: *Association between cell count or segmentation score and precision score. A. Association between number of cells in the test set and the respecting class AP. Each point is labeled with the class name. B. Average segmentation scores (mean IOU) did not correlate with mAP (Methods). Each point is labeled with team rank.*

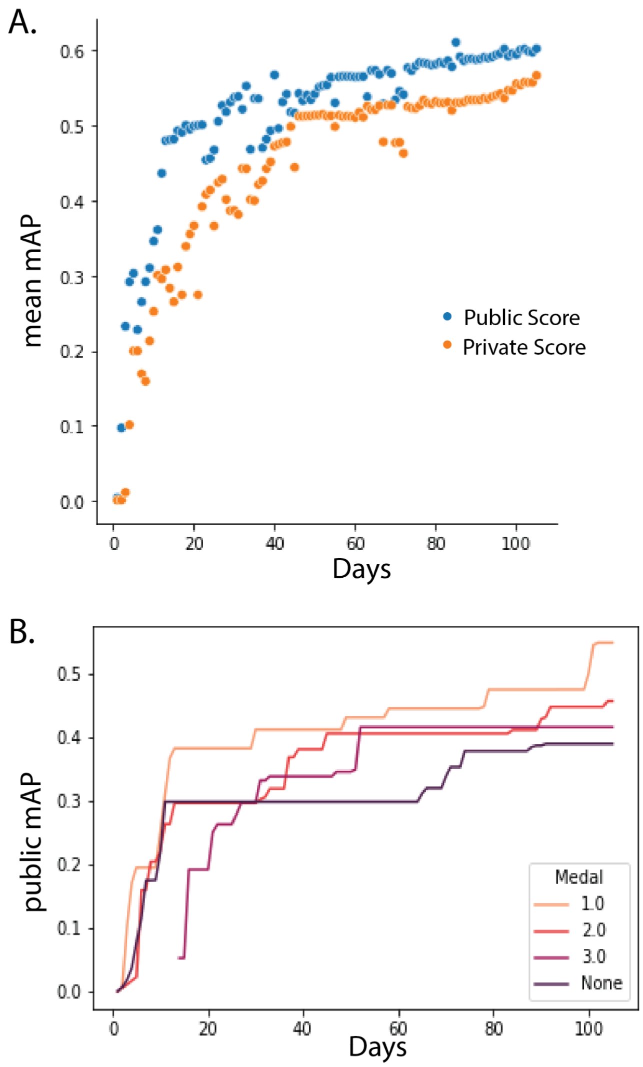

Supplementary Figure 2*: Score progression during the competition. A. Average of maximum mAP score that each team achieved so far on public and private leaderboard. B. Average score progression on the public leaderboard for all teams grouped by medals.*

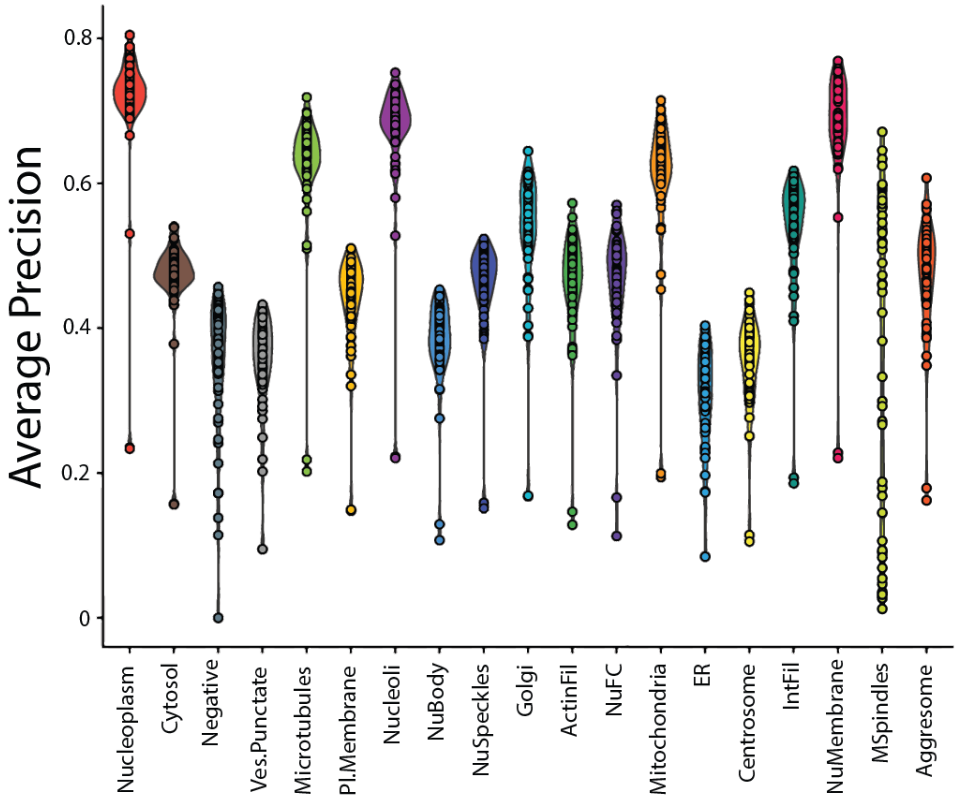

Supplementary Figure 3*: Violin plot of the score distribution per label for the top 50 teams, ordered by decreasing cell count. n=50 teams for each violin. The minimum, mean, percentile (P), and maximum values are: Nucleoplasm (min: 0.22, mean: 0.68, 25th P: 0.68, 50th P: 0.7, 75th P: 0.72, max: 0.77), Cytosol (min: 0.15, mean: 0.44, 25th P: 0.43, 50th P: 0.45, 75th P: 0.47, max: 0.51), Negative (min: 0.0, mean: 0.34, 25th P: 0.32, 50th P: 0.38, 75th P: 0.41, max: 0.47), Vesicles and punctate cytosolic patterns (min: 0.08, mean: 0.31, 25th P: 0.29, 50th P: 0.32, 75th P: 0.35, max: 0.39), Microtubules (min: 0.21, mean: 0.63, 25th P: 0.63, 50th P: 0.65, 75th P: 0.67, max: 0.71), Plasma membrane (min: 0.14, mean: 0.42, 25th P: 0.41, 50th P: 0.44, 75th P: 0.47, max: 0.5), Nucleoli (min: 0.21, mean: 0.63, 25th P: 0.62, 50th P: 0.64, 75th P: 0.67, max: 0.7), Nuclear bodies (min: 0.1, mean: 0.36, 25th P: 0.34, 50th P: 0.37, 75th P: 0.4, max: 0.44), Nuclear speckles (min: 0.18, mean: 0.57, 25th P: 0.56, 50th P: 0.59, 75th P: 0.61, max: 0.65), Golgi apparatus (min: 0.15, mean: 0.51, 25th P: 0.49, 50th P: 0.52, 75th P: 0.56, max: 0.61), Actin filaments (min: 0.14, mean: 0.48, 25th P: 0.46, 50th P: 0.49, 75th P: 0.53, max: 0.59), Nucleoli fibrillar center (min: 0.13, mean: 0.5, 25th P: 0.48, 50th P: 0.52, 75th P: 0.54, max: 0.58), Mitochondria (min: 0.17, mean: 0.55, 25th P: 0.54, 50th P: 0.56, 75th P: 0.6, max: 0.7), Endoplasmic reticulum (min: 0.06, mean: 0.25, 25th P: 0.21, 50th P: 0.25, 75th P: 0.31, max: 0.35), Centrosome (min: 0.09, mean: 0.33, 25th P: 0.31, 50th P: 0.36, 75th P: 0.37, max: 0.43), Intermediate filaments (min: 0.15, mean: 0.49, 25th P: 0.47, 50th P: 0.51, 75th P: 0.54, max: 0.57), Nuclear membrane (min: 0.22, mean: 0.68, 25th P: 0.66, 50th P: 0.69, 75th P: 0.73, max: 0.77), Mitotic spindle (min: 0.01, mean: 0.38, 25th P: 0.13, 50th P: 0.47, 75th P: 0.59, max: 0.69), Aggresome (min: 0.19, mean: 0.52, 25th P: 0.51, 50th P: 0.54, 75th P: 0.56, max: 0.66).*

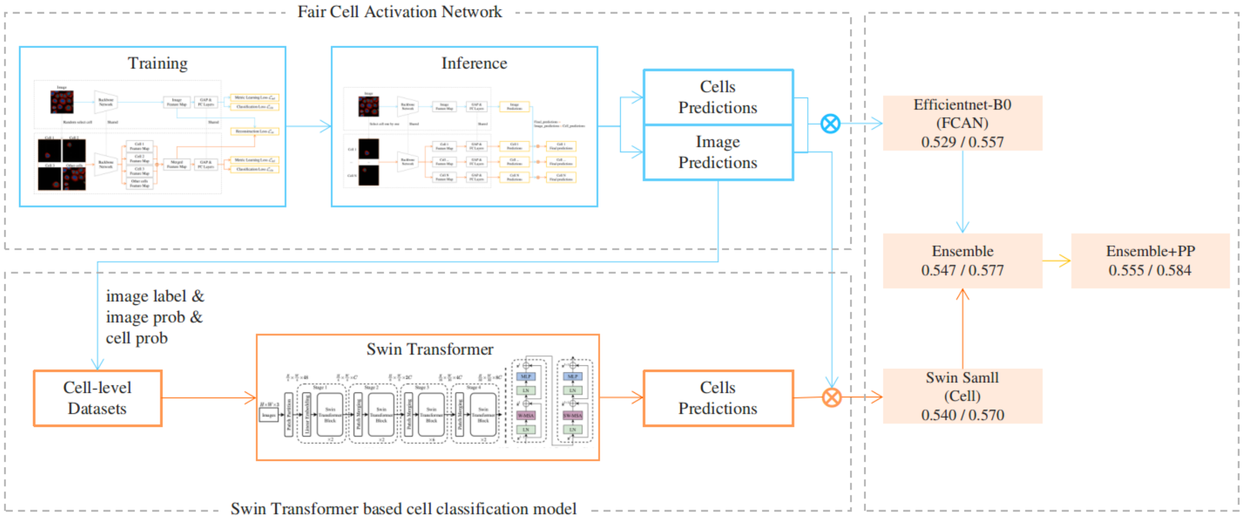

***Supplementary Figure 4**: Team 1 (bestfitting) - Solution summary and model architecture. Overview of the pipelines for the final solution, which include pipelines for cell-level and image-level prediction (Fair Cell Activation Network or FCAN) and subsequent cell prediction (Swin Transformer). The FCAN (blue) was trained on image-level labels and output image and cell probabilities for each class. Pseudo-cell-level labels were determined by these results, and used as training targets for the cell-level models (orange, in this case Swin Transformer) to predict the final single-cell predictions.*

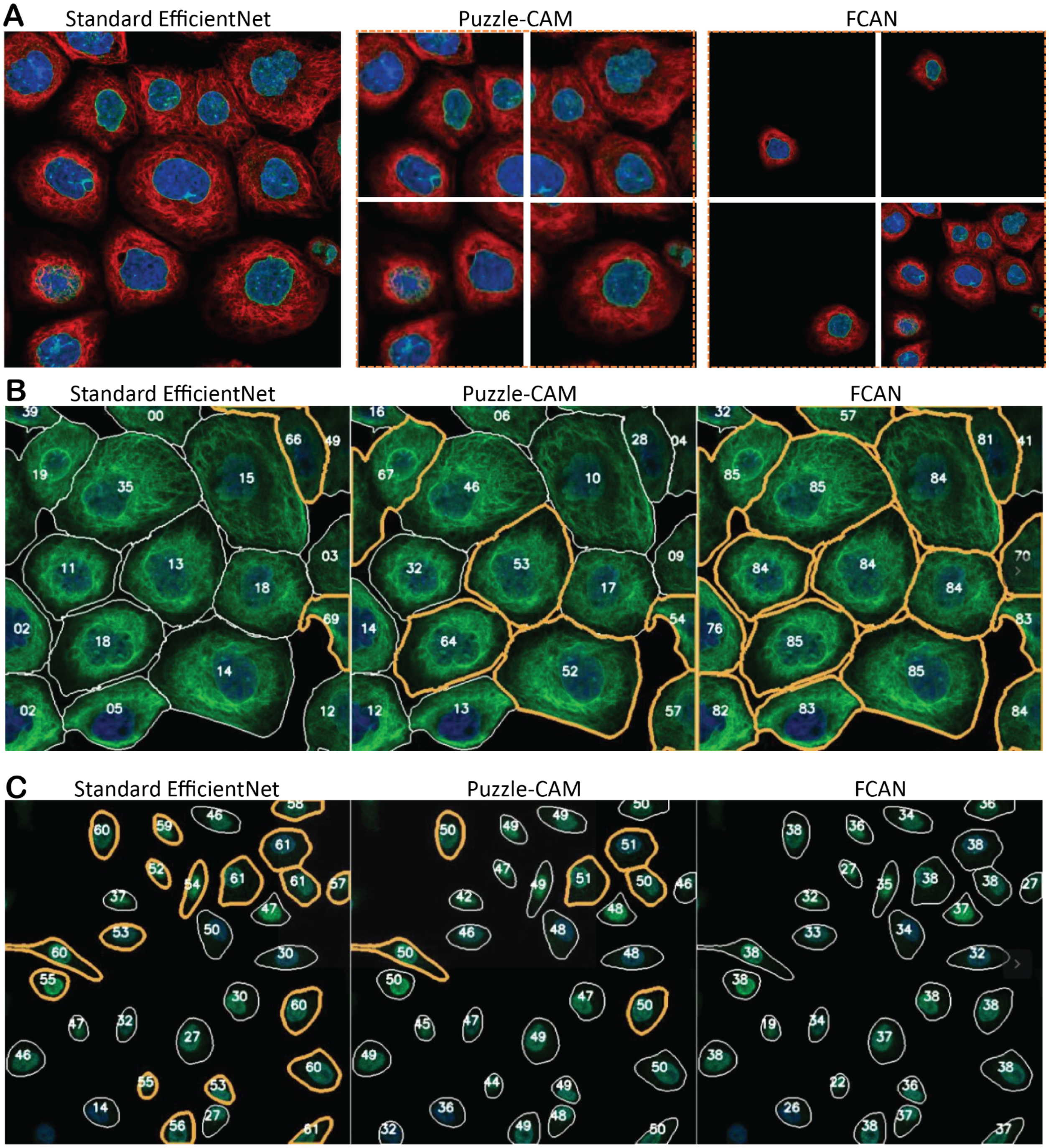

Supplementary Figure 5*:* *Team 1 (bestfitting) - Comparison between traditional convolutional network - EfficientNetB0 (Public AP 0.479, Private AP 0.492), Puzzle-CAM (Public AP 0.480, Private AP 0.508) and FCAN (Public AP 0.529, Private AP 0.557). A. Inputs of EfficientNet-b0 (whole image), Puzzle-CAM with EfficientNet-b0 backbone (random cropped patches) and FCAN with EfficientNet-b0 backbone (random cropped masked patches). B. Nuclear membrane prediction comparison: increasingly more cells were predicted with labels from EfficientNet to Puzzle-CAM to FCAN. C. Negative example comparison: increasingly fewer cells were predicted with labels from EfficientNet to Puzzle-CAM to FCAN.*

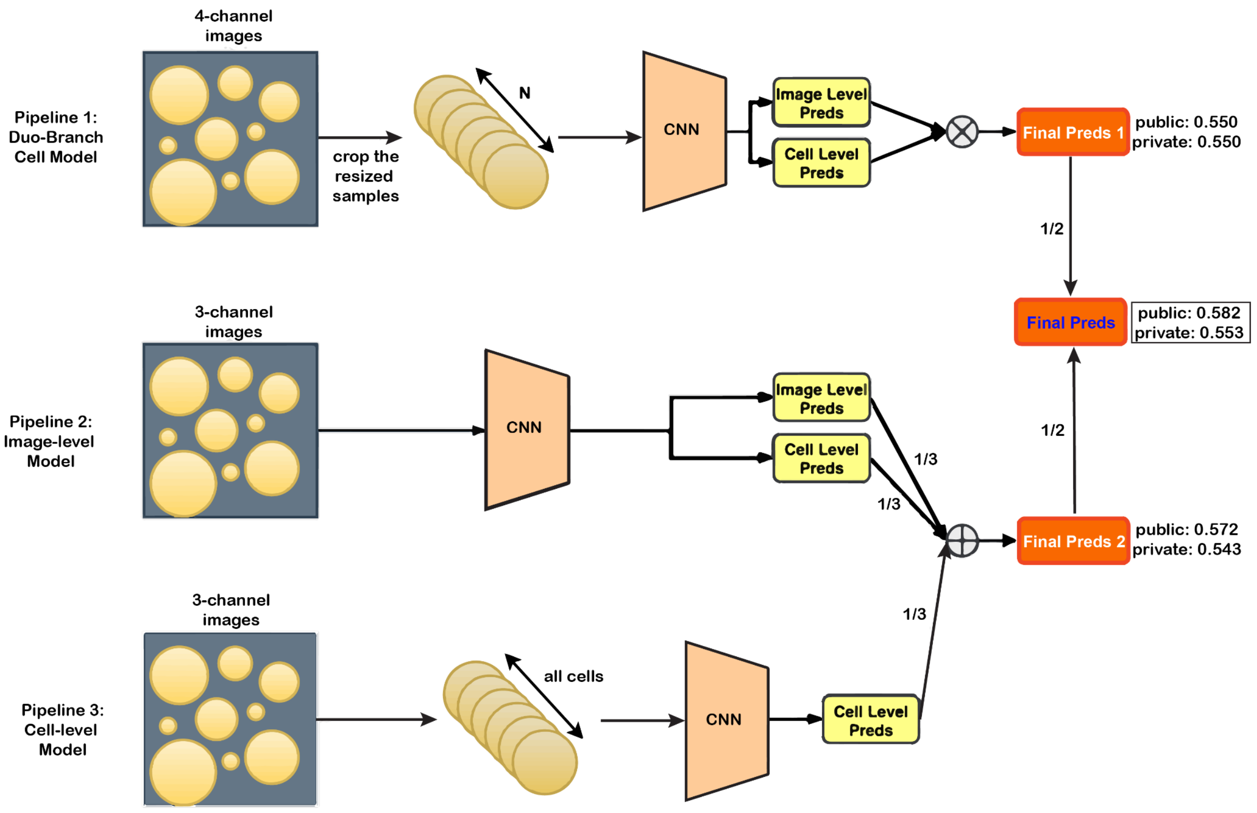

***Supplementary Figure 6**: Team 2 ([red.ai]) - Solution summary and model architecture. The final solution was the ensemble of 3 pipelines: duo-branch models which gave image-level and cell-level predictions, image-level models which gave image-level and cell-level predictions, and cell-level models which gave cell predictions.*

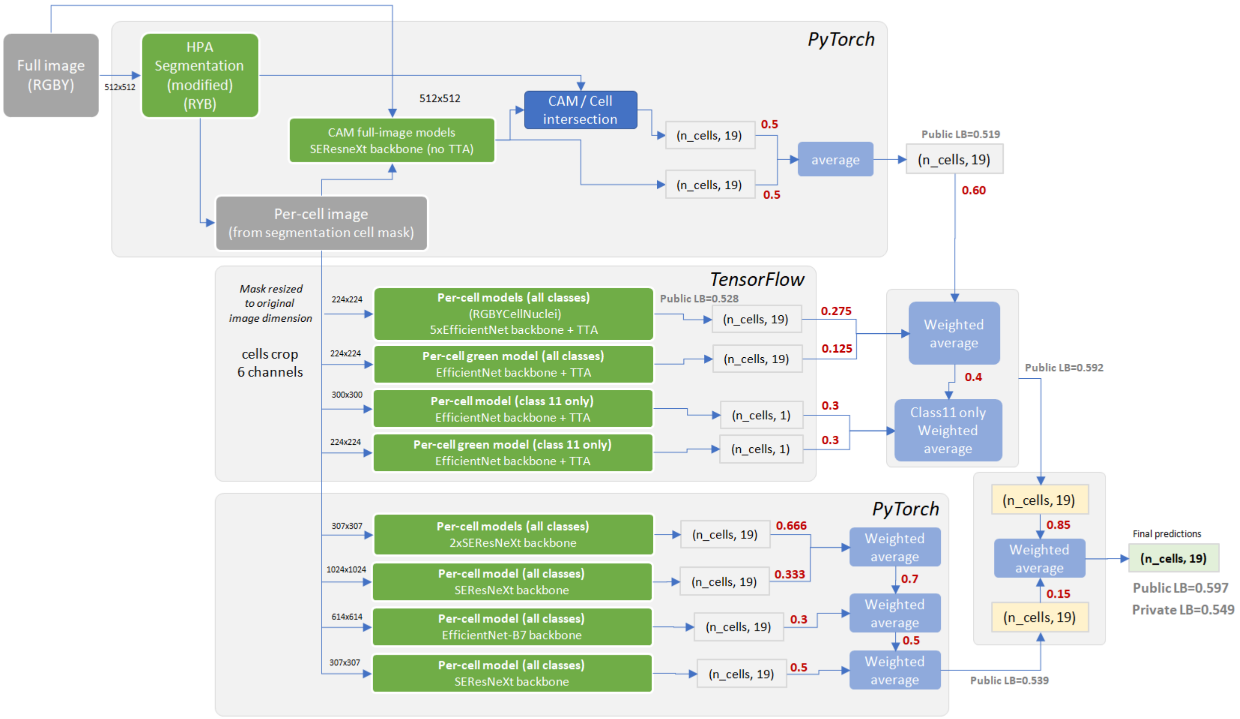

***Supplementary Figure 7**: Team 3 (MPWARE & ZFTurbo & Dieter) - Solution summary and model architecture. The final approach consisted of different models, broadly categorized as Image-level models and cell-level models. For image-level methods, full images were given as network inputs and the models were trained to find all the cells and their classes. Cell-level methods intaked single cells and predict the cell labels.*

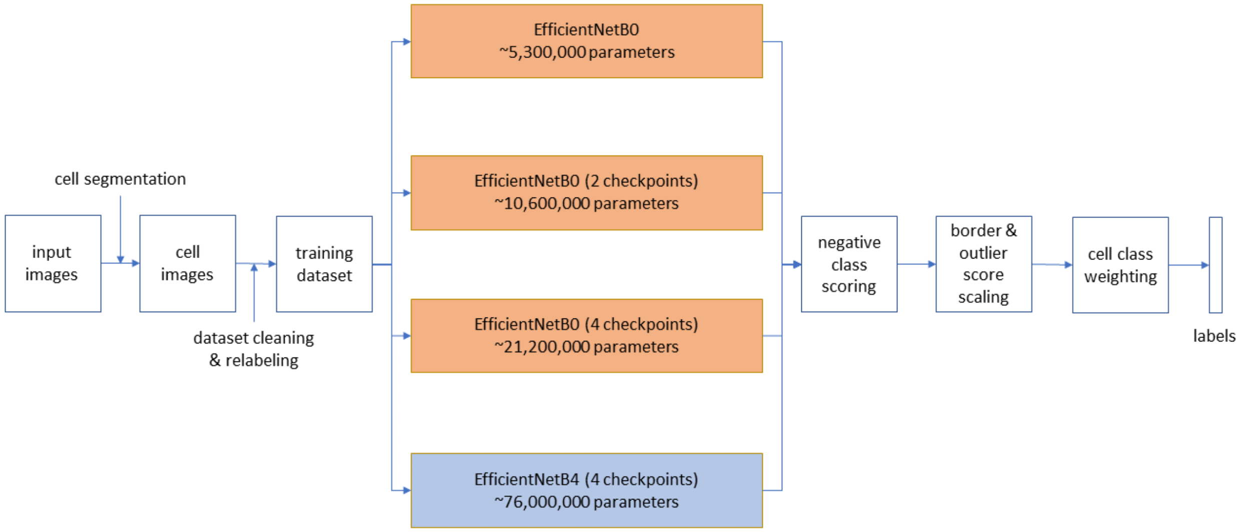

Supplementary Figure 8*: Team 4 (MILIMED) - Solution summary and model architecture. The solution consists of an ensemble of different cell-level models. Instead of model-centric approaches of other teams, MILIMED employed a data-centric approach, focusing on creating a better dataset with more accurate cell-level labels that would enable even simpler models to achieve highly accurate performance.*

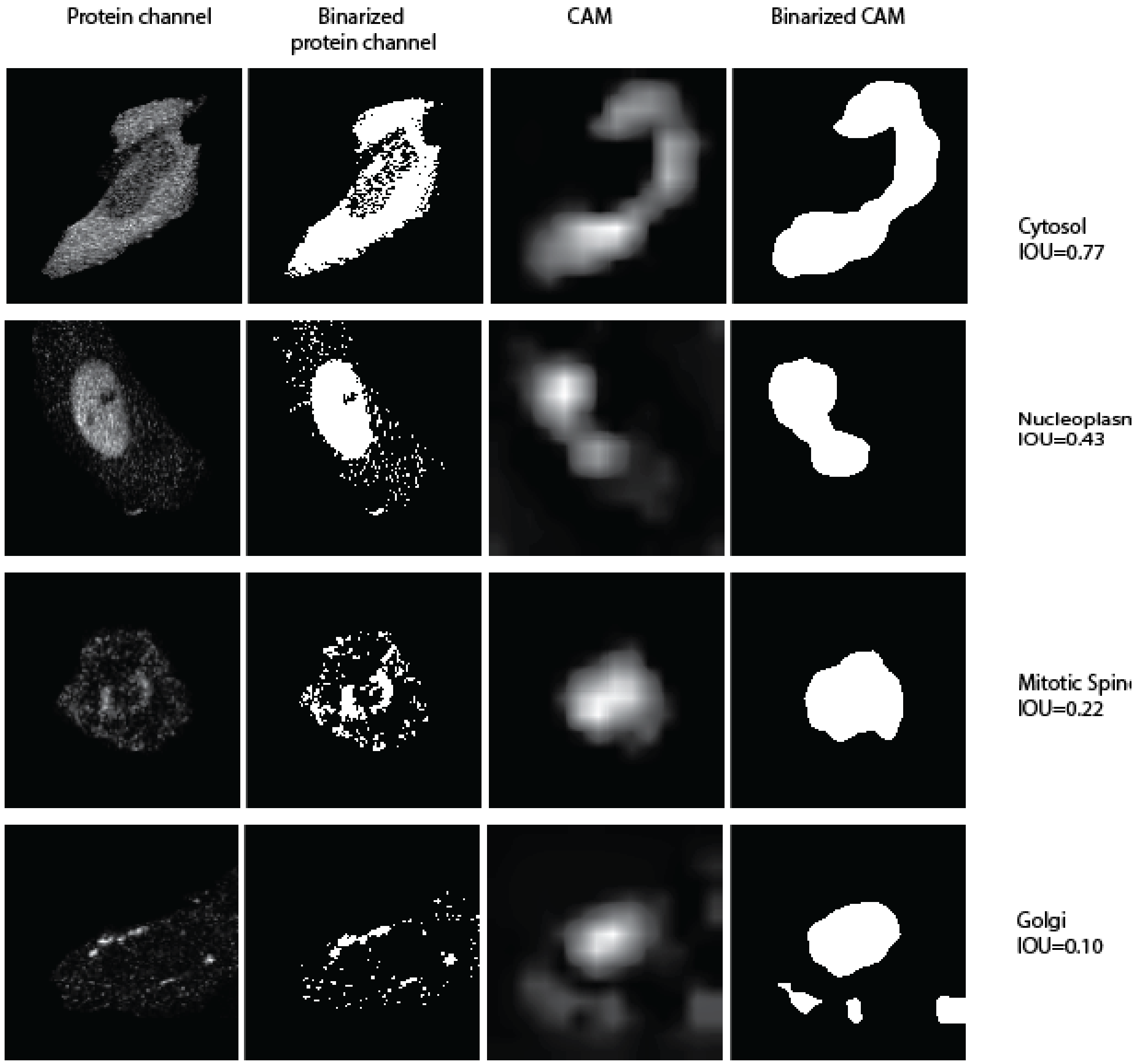

Supplementary Figure 9*. Class attention comparison to signal in green channel: intersection over union within the cell mask of binarized class activation and binarized protein signal.*

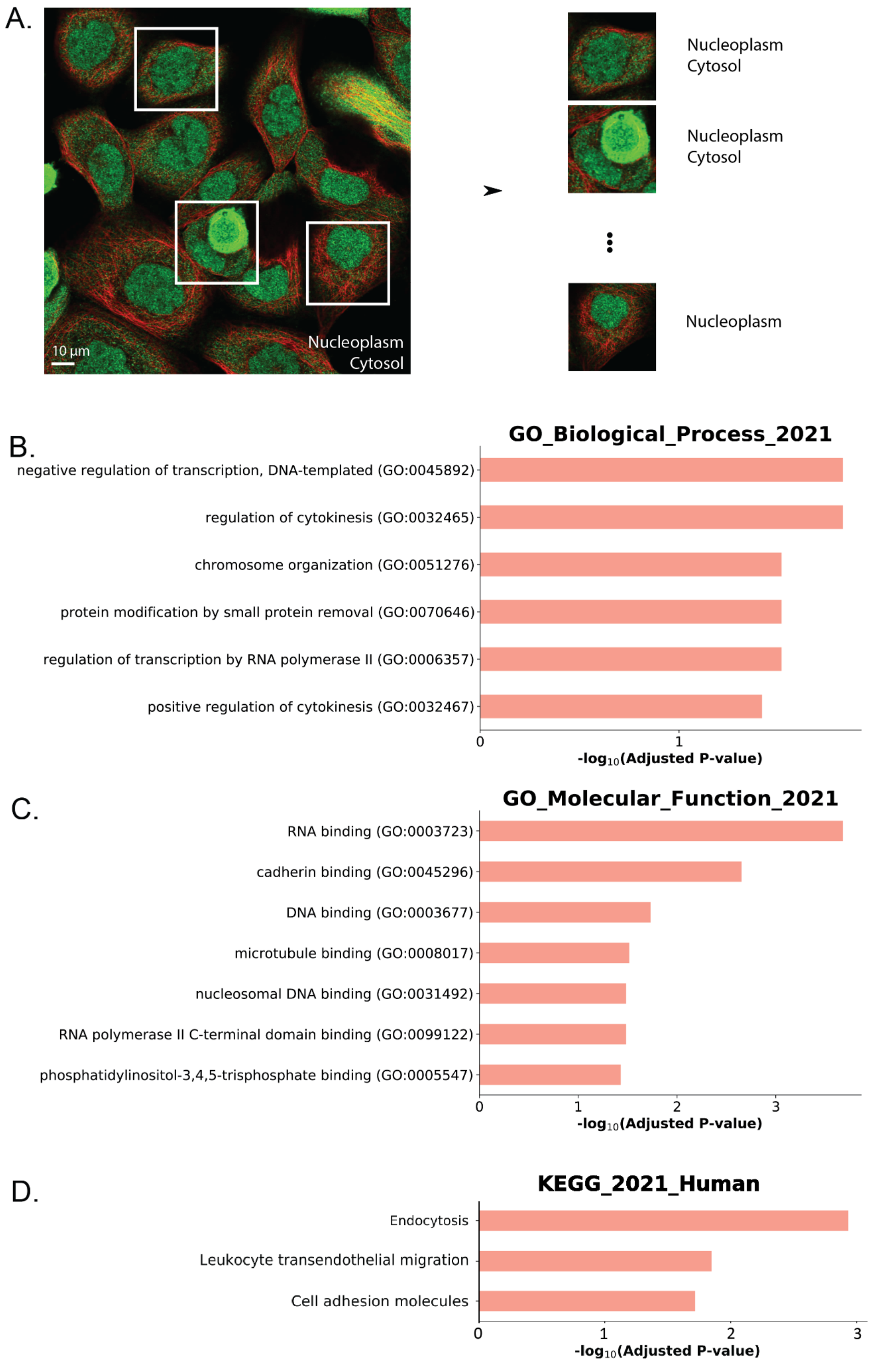

Supplementary Figure 10. *Subtle single cell heterogeneity discovered by the single cell model. A. An example of subtle heterogeneity. ZNF195 expression in A-341 cell line is annotated in Nucleoplasm and Cytosol. While most cells seem to express these two patterns, some cells only expressed Nucleoplasm. B,C,D. Gene set enrichment analysis of 452 proteins in GO_Biological_Processes, GO_Molecular_Functions and KEGG at Benjamini-Hochberg adjusted p-value of 0.05 (two-sided). There is enrichment in DNA binding functions, which is consistent with the overlap with CCD proteins.*

Supplementary Notes 1. Team 1 - bestfitting

1.1 Model description

The final solution (Supplementary Figure 4) was an ensemble of two networks: a Fair Cell Activation Network trained on image-level annotations and predict image-level and single-cell label probabilities, and a transformer network trained on thresholded cell-level predictions by FCAN. This ensemble and post-processing (especially reducing confidence for border cells) achieved AP 0.566/0.590 on the private/public leaderboard respectively.

Image-level model - Fair Cell Activation Network

The activations of convolutional neural networks (CNN) on feature maps of an image focus on most discriminative instances of a class despite many instances exist. To address this phenomena of unfair activation, a network called Fair Cell Activation Network (FCAN) was proposed. There are two branches in FCAN. The first branch took the full image as input and predicted the labels of this image (image classification task). Four images are fed into the second branch, three of them are cells randomly selected from this image, and the last one contains the remaining cells. The feature maps of these images were calculated and merged into one feature map, and the final labels were predicted just as if they were from one image. The backbone is shared among these two branches. The network is trained with Reconstruction Loss, which forces the feature maps from these two branches to be as close as possible. The intuition behind this network is that if we input all the cells in a whole, the network will only activate most discriminative cells, if we get a feature map of every cell and merge them at the last stage of the network, we can get fairer activations.

Cell-level models

The cells were cropped from images using HPACellSegmentator and labeled to 5 levels with label [1.0, 0.75, 0.5, 0.25, 0], this is a rule based procedure: After getting the outputs of all the cells of a train set image from FCAN, we give higher label value if the image-level probability and cell-level probability are high, and we assign a label at least 0.25 if the label exists in the image level labels. The cells are resized to 128x128px or 224x224px to feed into cell-level networks, including inceptionv3[1], DeiT[2] transformer and Swin[3] transformer.

Ensemble

Final probabilities for single-cell classes are the weighted averages of the predictions from FCAN and cell-level models.

Post-Processing

According to the setting of this competition, some cells on the border were not labeled. If these cells were predicted with high confidences, the False Positive cells will increase, so a model was trained to predict the completeness of a cell. If the probability to be a whole cell is very low, the confidence of this cell is multiplied by a low value such as 0.3.

1.2 Ablation study

Single models and experiments were reported in Supplementary Table 6.

Experiment #1-13 are experiments of FCANs. #2 shows that adding mitotic spindles with high confidence to other images to generate more positive samples of this type can improve the score. #3 tried to feed more cells to the FCAN model and the score decreased. #5 trained the model by adding external data which led to a better model as expected. We replaced Efficient-B0[4] backbone with more complex ones such as seresnet152[5] in experiments #6 #7 #8 and found no improvements. #9#10#11 suggested that bigger and deeper EfficientNet[4] models did not help performance, particularly in the public leaderboard. #12 #13 shows EfficientNet-B5 and EfficientNet-B7 decrease the score.

Experiment #14-22 are experiments of cell-level models. In experiments #15, #16, #17, we tested different image sizes and the results suggest that using larger images does not mean better score on the Swin[3] Small model. #20 #21 shows large images can improve the score on the Deit[2] Small model. #23 #24 compared the results before and after border-cells post processing, we can find that the border-cell model can improve the score significantly.

1.3 Conclusion

- Image-resolution had little impact on the final score.
- The Fair Cell Activation Network can increase cell level recall rate which is very important to this competition.
- The vision transformer models have shown promising capability.
- Larger models do not always mean better results as our models should find patterns of relative positions of pixels instead of abstract semantics.
- Data augmentation and Post-Processing are important.

Supplementary Notes 2. Team 2 - [red.ai]

2.1 Model description

Our solution is an ensemble of 3 convolutional neural network (CNN) pipelines: duo-branch cell pipeline (weight 0.5 in the final prediction), image-level pipeline (0.334 in the final prediction) and cell-level pipeline (0.167 in the final prediction). To extract single cells from the original image, HPACellSegmentator[6] with some modifications that formalize the manual annotation workflow was used. In particular, all bordered cells whose nuclei's areas are less than half the median area of all the non-border nuclei in the same image were removed.

Each pipeline consisted of ensemble of different single models described below:

Pipeline 1 - Duo-branched cell models

Cell tiles are first produced by cropping 16 random cells from the original 4-channel image, resizing them to 256x256 and then stacking them 4x4 to form a 1024x1024 image. If the image has less than 16 cells, the missing cell tiles are replaced with black tiles. The pipeline takes these cell tiles as input and predicts labels for all cells present on the combined tiled image (image-level) and for single cells (cell-level). Prediction for every cell is a product of the predictions of the two levels. The loss function used is a combination of cross-entropy losses with weights 0.1 for the image-level and 1 for the cell-level labels. Augmentations on tiled images applied during training included dihedral, shift, rotate, scale, distortions, brightness contrast and cutout. When predicting, an average of 16 test-time augmentations (TTA) was used (scale, rotate, flip at random). In this pipeline the following backbone architectures were trained: EfficientNet-B3, EfficientNet-B5 (both with noisy student weights)[4], ResNet200d[7], SEResNext50[5] - resulting in 64 predictions (4 backbones x 16 TTA) for every initial image.

Pipeline 2 - image-level pipeline

This pipeline consists of two parts, a and b.

a) the original RGB images with masked border cells as described above (red - microtubules, green - protein of interest, blue - nucleus, no yellow - endoplasmic reticulum was used) were resized to 512x512 and the labels for these resized images were predicted (multi-label classification). Weighted cross-entropy loss was used with class weights: 0.1, 1., 0.5, 1., 1., 1., 1., 0.5, 1., 1., 1., 10., 1., 0.5, 0.5, 5, 0.2, 0.5, 1. for class 0-19. Five architectures (EfficientNet-b5 with noisy student weighs[4], ECA-ResNet50t[8], EfficientNet-v2-small[8,9], ECA-NFNet-L0[8,10], ECA-NFNet-L1[8,10]) with 2-fold data splits were trained. Finally, an average of the predictions of 10 models (2 folds x 5 architectures) was used as the final prediction for a. This prediction contributed 0.167 in the final submission.

b) For every cell in an image, all other cells were masked producing a masked image which is resized to 512x512. The masked images are fed into the above 10 models. The predictions for every cell were averaged and this average contributed 0.167 in the final submission.

Pipeline 3 - cell-level pipeline

Every cell was cropped from the original RGB image, resized to 168x168 and then labels were predicted (multi-label classification). When training, the labels from the original image were assigned to each cropped cell. Weighted cross-entropy loss was used with class weights: 0.1, 1., 0.5, 1., 1., 1., 1., 0.5, 1., 1., 1., 10., 1., 0.5, 0.5, 5, 0.2, 0.5, 1. for class 0-19. Ten architectures (EfficientNet-b5 with noisy student weights[4], ECA-ResNet50t[8], EfficientNet-v2-small[8,9], dm-NFNet-F0, dm-NFNet-F1, dm-NFNet-F2, dm-NFNet-F3[8,10], SEResNet-152d[11], ECA-ResNet50d[12]) without splitting the data into folds were trained. In this pipeline an average of the predictions of the 10 models (1 fold x 10 architectures) was used as the final prediction.

2.2 Ablation study

Single models and experiments were reported in Supplementary Table 7.

The main finding of our experiments is that the ensemble of the models both between the pipelines and within every pipeline had the highest effect on the private LB score. More specifically, the highest LB score for a single pipeline (pipeline 1) was 0.55011 (Experiment #7) while combining the predictions from all the three pipelines increased the score to 0.57. Combining the predictions from pipeline 2 and 3 increased the score from 0.50871 (pipeline 2 alone, #8) to 0.51853 (pipeline 2 and 3 combined, #15). Finally, in pipeline 3 the single models scored between 0.470 and 0.487 (#13) while the averaging of all the predictions from this pipeline increased the score to 0.50533 (#12).

Because the final submission consisted of many models in combination with test-time augmentations, it was interesting to evaluate the performance of fewer models in the pipelines. When only one model of every pipeline was used without any test-time augmentations, the score dropped to 0.53698 (# 16) which is higher than a score for every used model. This further supports the finding that ensembling is the key for high score.

Apart from ensembling, the use of external HPA data, especially for the rare classes, was important for score improvement. For example, comparing #5 and #6 one can see that in pipeline 1 the use of external data improved the public leaderboard score by 0.044 and private leaderboard by 0.009. When training models for pipeline 3, we found that training for more epochs always led to overfitting and decrease in score. Most likely, this is due to bias from image-level labels that got assigned to cells and no true labels for a single cell were available.

2.3 Conclusion

- The ensemble of the models both between the pipelines and within every pipeline had the highest effect on the private LB score.

Supplementary Notes 3. Team 3 - MPWARE & ZFTurbo & Dieter

3.1 Model description

Our solution consists of different methods from each of 3 team members. These methods can be divided into 2 big classes: “image-level” and “cell-level”. For image-level methods, full images were given as neural network (NN) inputs and the models were trained to find all the cells and their classes. Cell-level methods intaked single cells and predict the cell labels.

Image-level models:

Image-level models were trained with ~98K images. These models’ architectures were similar to PuzzleCAM[13]. We used a Siamese network[14] with CNN backbone followed by a classifier to build activation maps per class. Global Average Pooling (GAP) and classifier were swapped to get full image predictions. Combined loss (1.0 x BCELoss + 0.25 x L1Loss) was used. The models took into account both full and recomposed features as well as the distance between activation maps. The inputs to the Siamese network were full RBGY images resized to 512x512 and each part of the same image splitted into 4 chunks. Weighted sampling was used to combat extreme class imbalance. Main training steps were:

● Cross Validation: Train dataset was split in 4 folds with a MultilabelStratifiedKFold strategy

● Weighted random sampling to balance each class during training: Common classes like Nucleoplasm were given less weight than rare classes like Mitotic spindle. Oversampling were done for rare classes 11 and 15, with clipped weights value to what computed on Intermediate filaments (class 8).

● Augmentations on RGBY images: GaussNoise, CoarseDropout, IAAAdditiveGaussianNoise, HorizontalFlip, RandomRotate90, GridDistortion, ShiftScaleRotate, ElasticTransform, OpticalDistortion, IAAAffine/Shear, GaussianBlur, MotionBlur, MedianBlur, RandomGamma, RandomBrightnessContrast.

● Optimizer: Adam (LR=0.0003, beta1=0.9), LR scheduler: ReduceLROnPlateau, (factor = 0.3, patience = 8).

● Half precision was used to reduce memory consumption and increase batch size.

● Epochs: 48, batch size: From 32 to 36.

● Best ComboLoss only was the criteria to save the model's weights.

Inference consisted of two stages. Predictions at each stage were ensembled at the end for final prediction.

● Stage 1: Class activation maps (CAM) from the trained model were normalized to [0,1] and resized to input image size. HPACellSegmentator was executed in parallel to get cell masks. Each cell is intersected with CAM (overlap + magnitude score) and then weighed with the per-class probability outputted from the sigmoid layer.

● Stage 2: Single cell patches were inputted to the same pretrained image-level model to predict cell probabilities.

Predictions from stage #1 and stage #2 were different by design: Stage 1 are more sparse whereas stage 2 are more flatten. Both acting together gave some regularization to the results.

Simple cell-level models

The training of cell-level models was straight-forward. Image-level labels were assigned to each independent cell on the image. The images were KFold splitted using the cell line information for external data. Compared to random split, splitting by cell line resulted in larger differences between the validation and training data, forcing models to have better generalization to have higher validation score. Training on all available data or only external data without the official training set gave very similar results.

Backbone models included different EfficientNets[4]: EfficientNet-B0, EfficientNet-B3 and EfficientNet-B5. The quality for all of them was similar. We used sigmoid on the final layer with BCE loss and later switched to Focal Loss which observed slight score improvement. We also used soft labels with 0.01*num_labels coefficient. Large dropout (0.5) was added to prevent overfitting. Single cell 6-channel images [red, green, blue, yellow, mask, nuclei] were provided as inputs for most models. Some models were trained only on a single green channel. Various augmentations were used, including random crops, rotations, etc. At the inference stage, test time augmentation (TTA2) – original and mirror image - were used. TTA8 (8 augmentations) could improve the score, though time-consuming.

Several metrics were used for validation: Avg AUC per class, Avg Accuracy per class and LogLoss. ReduceLROnPlateu for AUC metrics was mainly used for early stopping. After some point the model tends to overfit.

Simple cell-level models had typical scores from 0.460 to 0.480 on the public leaderboard, and around 0.465 on the private leaderboard. Because of the weak label challenge, cell-level model approach observed no improvements after some point. There were great discrepancies between cell labels and image labels for multi-labeled images, especially for rarer classes. By training on image-level labels, models gave high probability for incorrect classes, which meant increasing the number of false positives over time. Attempts at training on single class images didn’t give an additional boost in score.

Cell-level models trained on OOF predictions

KFold split was used for all our models. Therefore, it was possible to create out-of-fold (OOF) predictions for all training data and all external data. This approach was applied for some simple cell-level models as well as for image-level models. Ensemble of these models on test data got a great score boost, therefore we expected that labels obtained for independent training cells would become better for ensemble of OOF predictions. Mixed OOF predictions were ensemble similarly to the ensemble of our models for submission. This way we created markup which was closer to “ground-truth” e.g. decrease weakness of cell labels. Using these new cell-level labels

obtained by ensembling OOF predictions as new target, we trained the cell-level models as described previously. Mean square error loss was minimized during.

These models give around 0.528 on public and private leaderboards. The same models were trained using green channel only. These two cell-level models, trained on OOF, were included in our final ensemble instead of simple cell-level models.

While creating OOF data, because cells were extracted with slightly different algorithms, it wasn't always possible to find the same cell in predictions of different models. This resulted in around 90% of cells being included in OOF with some small noise.

Single class cell-level models

Class 11 (Mitotic spindle) had the worst performance particularly on cell-level models, partially because of the low amount of images available and extremely high single cell variation (1/20 cell in an image actually has this label). Therefore, we spent some time labeling for this class manually. A binary classifier model was trained with these manual labels, and the score increased +0.008 on public and +0.014 on private leaderboard.

3.2 Ablation study

Single models and experiments were reported in Supplementary Table 8. This ablation study aims to understand how the data, validation strategies, loss functions, and network structures affected the model capabilities.

● Experiment for cell-level models are #1 - #8, all used EfficientNetB5 with image size 224x224. From default training data and single fold (0.402, #1), the score improved when adding 5 KFold split (0.421, #2). External data also improved the score for single fold (0.438, #3) and 5 Kfold split (0.444, #4). Splitting based on cell line information, which was available only for external data, achieved a similar score on public leaderboard (0.443, #5). Reducing cropped cell regions increased the score to 0.476 (#6). Finally, TTA8 (8 combinations of rotation and mirror of an image) increased the score marginally compared to TTA2 (original image and horizontal mirror) from 0.476 (#6) to 0.47 (#7), but increased inference time by 4 times. Therefore, later experiments used TTA2.

● Experiments with higher regularization by dropout (0.5 vs 0.25) using a similar cell-level model (EfficientNet-B0) are #7 (0.470) vs #9 (0.471) and #6 (0.476) vs #10 (0.465).

● Adding a 6th channel with a nucleus mask as input data (#11) improved the score slightly on public (0.470 -> 0.471), but more on private (0.450 -> 0.457). Scores for EfficientNet-B5 and EfficientNet-B0 were almost the same, but the number of parameters for B5 was much larger. Therefore, smaller EfficientNet models, mostly B3 and B0, were used to save inference time.

● Comparison of different losses and soft labels for cell-level models: Training with different parameters (BCE loss and Focal loss with usage of soft labels in experiments #12, #13, #14) led to almost the same results. Model trained only on single class images got much lower score (0.416, #15) on the test set, as it assumed the homogeneity of cells in the same image.

● Manual labels gave a great boost in score (0.012-0.03) for Mitotic Spindle compared to training on weak image-level labels.

● Experiments #19 to #20 showed that using either part of the siamese network on inference made little difference. Deeper backbone (#19, #20) showed slightly better results (#21).

● A large ensemble with many parameters can be compressed using OOF prediction to a smaller single model with very little loss of accuracy as shown in #23 and #24.

3.3 Conclusion

- Ensemble of cell-level and image-level predictions gives a great boost for quality of predictions.
- It's possible to compress a large ensemble of models with a small single cell-level model which was trained on out-of-fold predictions. Furthermore, models trained on OOF have much closer public and private scores. This insight is highly relevant for deploying an efficient model in production, as drastic reduction in the number training parameters could yield roughly the same score.
- The model trained only on green channels had slightly higher performance compared to RGBY models. It's possible that in some cases the models were confused by the RBY context.
- Class 11 had bad quality predictions because it was rare and very heterogeneous. Image-level models missed this class often while cell-level models sometimes see class 11 in other classes because of weak labels. Additional hand-labels created for this class helped for overall performance of the final solution.

Supplementary Notes 4. Team 4 - MILIMED

4.1 Model description

Cell-level models

This competition consisted of a dataset with weakly labeled images wherein every image contained multiple cells. Using image-level labels as cell-level labels was reasonable for classes, such as the nucleoplasm class, where this approach wouldn’t generate many false positives. But, for some classes, such as the Mitotic Spindle class, this approach would be extremely inaccurate since most of the generated cell-level labels would be false positives. Additionally, the dataset was highly imbalanced. Therefore a data-centric approach to this challenge (in contrast to the classic model-centric approach) was pursued. The main goal was to create a better dataset with more accurate cell-level labels that would enable even simpler models to achieve highly accurate performance.

The training dataset was created using the 16-bit competition and public HPA images. Cells were segmented using the HPACellSegmentator, which generated nuclei and cell masks. All cell images were zero-padded to retain original height to width ratio and then resized to 512x512. All 4 channels were used.

Two heuristics were used for dealing with border images and outliers. Based on the nuclei segmentation masks, calculations were made for each cell to approximate how much of the cell area is outside of the field of view. Using the cell segmentation masks, sums of cell input values were calculated for red, blue and yellow input channels. Outliers were detected by comparing the red channel sums and the product of the blue and yellow channel sums of each cell with a thresholded average of all cells from the same image. Cells that were discarded by the first heuristic were ignored when calculating the average values.

To create a clean dataset, rigorous thresholds of the mentioned heuristics were used. The heuristics had a precision of roughly 50%, but a very high recall. In the end, around 20% of the cell images generated by the mentioned segmentator were removed from the final training dataset. Since there was an abundance of cell images, data quality was prioritized over data quantity.

A GUI was created for fast manual relabeling and consequently around 150 000 cell-level labels were manually graded, i.e. relabeled. More specifically, positive labels were usually graded with scores on a scale from 1 to 5, which reflected the confidence that the positive label is correct. This was done for classes that were harder to predict. These scores were mapped to soft labels (e.g. 1 to 0.0, 2 to 0.2, 3 to 0.7, 4 to 0.9, and 5 to 1.0) which were used instead of the given image level labels. These soft label mappings were class specific. This process was done in a fast manner with the intention to focus on ruling out obvious incorrect cell-level labels.

Special care was taken for the mitotic spindle class. The mitotic spindle image-level labels matched cell-level labels in only ~3%, since the mitotic spindle is a structure that only appears in cells during division. All cell-level images that contained a positive mitotic spindle image-level

label were manually relabeled. Around 250 cell-level examples of the mitotic spindle class were found this way.

Since local validation was hard, a few thousand cell-level images were relabeled for most classes. This was done in a slower manner, but with higher precision. While this was a time-consuming process, it eventually led to better local validation. Around 30 000 cell-level labels were graded and again mapped to class specific soft labels.

With better local validation, it was possible to train better models and use them to further clean the current dataset. One ResNet18[11] with a single output was trained for each class separately. These models were then used to relabel almost all classes, but only positive labels were changed. Relabeling was done when the output of the ResNet18 model would be less than 0.3 for a label that was expected to be positive. Approximately 15% of the cells were relabeled this way. This has led to even better local validation.

By using the inverse approach, false negative examples of the mitotic spindle were detected. Around 100 examples of the mitotic spindle pattern cell-level images were relabeled this way.

All final models are from the EfficientNet[4] family. Three EfficientNet-B0 models as well as one EfficientNet-B4 model were trained using Adam as an optimizer with either focal loss or binary cross entropy loss. Best models were selected based on local mAP score. Checkpoint ensembling was used for most trained models. Different augmentation techniques were used for training: random resizing, random padding, flipping (horizontal and vertical) and rotation. After augmenting, images were resized to 512x512 when needed. Resizing images to a smaller size and random padding on each side was used to train models on different cell sizes and resolutions. This was inspired by the fact that training images differed in image resolution and cell size.

After ensembling trained models, negatives were calculated by subtracting the maximum output value from the value one. Next, border and outlier cells output prediction scores were decreased based on two previously mentioned heuristics. In the end, the final predictions were weighted with the average cell-level predictions of an image scene, separately for each output label (e.g. 0.7 * cell output + 0.3 * average image scene cell output). More details about the final submitted models can be seen on Supplementary Figure 8 and Supplementary Table 9.

4.2 Ablation study

Single models and experiments were reported in Supplementary Table 9. The ablation study showed that checkpoint ensembling had a negative effect on the final score, although showing a positive effect on local validation (#16, #22). All models were trained on 16-bit images. Converting all test images to 8-bit showed little impact on the final score (#5, #10, #15, #21). Cell weighting (#2, #7, #12, #18), border detection (#3, #8, #13, #19) and outlier detection (#4, #9, #14, #20) showed a positive effect on the final score. The biggest positive benefit was from ensembling models trained with different loss functions and augmentations.

4.3 Conclusion

Using the data-centric approach was the key component in the presented solution. Creating a better training dataset by using all available data, removing outliers and border images along with automatic and manual relabeling showed a bigger impact than training more complex models. The final dataset was still not perfectly labeled, which could explain the better generalization of simpler models in the presented approach. A single EfficientNetB0[4] model, trained on this dataset, resulted with a mAP score 0.53291 which alone would be enough for 11th place, while ensembling multiple models resulted with scores 0.54389 and 0.54361, both securing 4th place in this competition.

Supplementary Notes 5. Method summary

Solutions were assessed based on the following criteria and summarized in Table 2:

- Input and preprocessing
    - Pseudo-labelling:
    - Label-noise reduction:
    - Manual labelling of rare class
    - Thresholding:
    - Heavy augmentation
    - Image resolution: using high resolution (16bit) or not
- Segmentation
    - (enhanced) HPACellSegmentator: used or not
    - Segmentation postprocessing: use a separate (CNN) classifier for border cells
    - Edge heuristics: used heuristics to remove border cells
- Approach to handle single cell:
    - MaskRCNN: used or not
    - PuzzleCAM and modifications: used or not
    - Cell and image models combinations: used or not
    - Transformers: used or not
- Loss:
    - Weighed-loss/oversample: used weighed loss of oversampling to deal with imbalance
    - Focal loss: used or not
    - Combine 2+ losses: whether teams used a combination of multiple losses

References

1. Szegedy, C., Vanhoucke, V., Ioffe, S., Shlens, J. & Wojna, Z. Rethinking the Inception Architecture for Computer Vision. in *2016 IEEE Conference on Computer Vision and Pattern Recognition (CVPR)* 2818–2826 (IEEE, 2016). doi:10.1109/CVPR.2016.308.
2. Touvron, H. *et al.* Training data-efficient image transformers & distillation through attention. *ArXiv201212877 Cs* (2021).
3. Liu, Z. *et al.* Swin Transformer: Hierarchical Vision Transformer using Shifted Windows. *ArXiv210314030 Cs* (2021).
4. Tan, M. & Le, Q. V. EfficientNet: Rethinking Model Scaling for Convolutional Neural Networks. 10.
5. Hu, J., Shen, L. & Sun, G. Squeeze-and-Excitation Networks. 10.
6. *CellProfiling/HPA-Cell-Segmentation*. (CellProfiling, 2021).
7. He, T. *et al.* Bag of Tricks for Image Classification with Convolutional Neural Networks. *ArXiv181201187 Cs* (2018).
8. Wightman, R. *PyTorch Image Models*. (2022).
9. Tan, M. & Le, Q. V. EfficientNetV2: Smaller Models and Faster Training. *ArXiv210400298 Cs* (2021).
10. Brock, A., De, S., Smith, S. L. & Simonyan, K. High-Performance Large-Scale Image Recognition Without Normalization. *ArXiv210206171 Cs Stat* (2021).
11. He, K., Zhang, X., Ren, S. & Sun, J. Deep Residual Learning for Image Recognition. in *2016 IEEE Conference on Computer Vision and Pattern Recognition (CVPR)* 770–778 (IEEE, 2016). doi:10.1109/CVPR.2016.90.
12. Wang, Q. *et al.* ECA-Net: Efficient Channel Attention for Deep Convolutional Neural Networks. *ArXiv191003151 Cs* (2020).
13. Jo, S. & Yu, I.-J. Puzzle-CAM: Improved localization via matching partial and full features. *2021 IEEE Int. Conf. Image Process. ICIP* 639–643 (2021)

doi:10.1109/ICIP42928.2021.9506058.

14. Bromley, J., Guyon, I., LeCun, Y., Säckinger, E. & Shah, R. Signature Verification using a 'Siamese' Time Delay Neural Network. 8.
