# Supplementary material for: Analysis of the Human Protein Atlas Weakly Supervised Single-Cell Classification competition
Source: Nat Methods. 2022 Sep 29;19(10):1221–9. doi: 10.1038/s41592-022-01606-z (PMC9550622; doi:10.1038/s41592-022-01606-z)
Supplement: Supplementary file 2 — Reporting Summary [file 41592_2022_1606_MOESM2_ESM.pdf]

nature research

Corresponding author(s): Emma Lundberg

Last updated by author(s): Jun 28, 2022

Reporting Summary

Nature Research wishes to improve the reproducibility of the work that we publish. This form provides structure for consistency and transparency in reporting. For further information on Nature Research policies, see our Editorial Policies and the Editorial Policy Checklist.

Statistics

For all statistical analyses, confirm that the following items are present in the figure legend, table legend, main text, or Methods section.

n/a	Confirmed	
☐	☒	The exact sample size (*n*) for each experimental group/condition, given as a discrete number and unit of measurement
☐	☒	A statement on whether measurements were taken from distinct samples or whether the same sample was measured repeatedly
☐	☒	The statistical test(s) used AND whether they are one- or two-sided<br>*Only common tests should be described solely by name; describe more complex techniques in the Methods section.*
☒	☐	A description of all covariates tested
☐	☒	A description of any assumptions or corrections, such as tests of normality and adjustment for multiple comparisons
☐	☒	A full description of the statistical parameters including central tendency (e.g. means) or other basic estimates (e.g. regression coefficient) AND variation (e.g. standard deviation) or associated estimates of uncertainty (e.g. confidence intervals)
☐	☒	For null hypothesis testing, the test statistic (e.g. *F*, *t*, *r*) with confidence intervals, effect sizes, degrees of freedom and *P* value noted<br>*Give P values as exact values whenever suitable.*
☒	☐	For Bayesian analysis, information on the choice of priors and Markov chain Monte Carlo settings
☒	☐	For hierarchical and complex designs, identification of the appropriate level for tests and full reporting of outcomes
☐	☒	Estimates of effect sizes (e.g. Cohen's *d*, Pearson's *r*), indicating how they were calculated

Our web collection on statistics for biologists contains articles on many of the points above.

Software and code

Policy information about availability of computer code

Data collection	We used Leica SP5 confocal microscope (DM6000CS) equipped with a 63× HCX PL APO 1.40 oil CS objective (Leica Microsystems, Mannheim, Germany) and Leica Application Suite Advanced Fluorescence (LAS AF, 2.6.0 build 7266) to collect images used in this work. However the image collection (including experimental procedures and samples/reagents such as cell line authentication and antibody validation) is described in previous publications and is not part of this work.
Data analysis	For the competition dataset we mainly used Python 3 (with libraries such as numpy 0.17.0, pandas 0.24.2, scikit-image 0.15.0, scikit-learn 0.21.3, scipy 1.1.0, matplotlib 2.2.2, seaborn 0.9.0). Pretrained segmentation model is available at https://github.com/cellProfiling/hpa-cell-segmentation. Software libraries used by the top teams are described in their own source code available at https://github.com/topics/hpa-challenge-2021

For manuscripts utilizing custom algorithms or software that are central to the research but not yet described in published literature, software must be made available to editors and reviewers. We strongly encourage code deposition in a community repository (e.g. GitHub). See the Nature Research guidelines for submitting code & software for further information.

Data

Policy information about availability of data

All manuscripts must include a data availability statement. This statement should provide the following information, where applicable:

- Accession codes, unique identifiers, or web links for publicly available datasets
- A list of figures that have associated raw data
- A description of any restrictions on data availability

The dataset used for the HPA competition is available at: https://www.kaggle.com/c/hpa-single-cell-image-classification

The external dataset HPAv20 is publicly available on the Human Protein Atlas: https://v20.proteinatlas.org/
Dataset for training HPACellSegmentation model is available at: https://zenodo.org/record/4665863

Field-specific reporting

Please select the one below that is the best fit for your research. If you are not sure, read the appropriate sections before making your selection.

☒ Life sciences ☐ Behavioural & social sciences ☐ Ecological, evolutionary & environmental sciences

For a reference copy of the document with all sections, see nature.com/documents/nr-reporting-summary-flat.pdf

Life sciences study design

All studies must disclose on these points even when the disclosure is negative.

Sample size

Sample size of training and testing dataset, as well as public HPA was detailed in Figure 1A and Methods. For analysis, top 50 teams were chosen as representation of overall participating team's performance. Based on the available solution write-ups, we chose the top 4 teams as representation of the approaches in this competition.

Data exclusions

For training and testing datasets, inclusions and exclusion criteria were described in Methods.

For overall participation and performance analysis, all valid submissions were included. For subsequence class-specific analysis, as the size of the test dataset is large, many teams chose to submit either to public leaderboard (for method development and validation) or private leaderboard (for final ranking) only. Higher percentage of missing scores was observed in lower ranking team, therefore, all teams outside top 50 were excluded from the performance analysis.

Replication

As this is a code competition, all code submission for inference were collected and graded automatically, which allows for reproduction of the scores. Final ranking was determined after re-runing all team's chosen submissions.

All training data and HPAv20 are publicly available for model training and testing dataset is available on Kaggle for replication of the performance. Teams independently make their code base open-sourced on Github, especially for the top 4 teams.

Randomization

Assembling the test set was based on criteria described in Methods, but the individual images were sampled randomly. Annotation of cells in the test set was done individually, in random order and assigned randomly to annotators to avoid bias.

Blinding

The participants did not have access to any cell labels in all datasets as well as images from the private test set, ensuring a fair evaluation. As with code competition, scoring algorithm was performed automatically without manual intervention.

Reporting for specific materials, systems and methods

We require information from authors about some types of materials, experimental systems and methods used in many studies. Here, indicate whether each material, system or method listed is relevant to your study. If you are not sure if a list item applies to your research, read the appropriate section before selecting a response.

Materials & experimental systems

n/a	Involved in the study	
☒	☐	Antibodies
☒	☐	Eukaryotic cell lines
☒	☐	Palaeontology and archaeology
☒	☐	Animals and other organisms
☒	☐	Human research participants
☒	☐	Clinical data
☒	☐	Dual use research of concern

Methods

n/a	Involved in the study	
☒	☐	ChIP-seq
☒	☐	Flow cytometry
☒	☐	MRI-based neuroimaging
